# The magnitude of local adaptation under genotype-dependent dispersal

**DOI:** 10.1002/ece3.850

**Published:** 2013-10-30

**Authors:** Daniel I Bolnick, Sarah P Otto

**Affiliations:** 1 Howard Hughes Medical Institute, Department of Integrative Biology, University of Texas at AustinOne University Station C0990, Austin, Texas, 78712, USA; 2 Department of Zoology, University of British Columbia6270 University Blvd., Vancouver, BC, V6T 1Z4, Canada

**Keywords:** Fitness-associated dispersal, genetic load, habitat choice, habitat selection, local adaptation, matching habitat choice, migration-selection balance, natural selection

## Abstract

Dispersal moves individuals from patches where their immediate ancestors were successful to sites where their genotypes are untested. As a result, dispersal generally reduces fitness, a phenomenon known as “migration load.” The strength of migration load depends on the pattern of dispersal and can be dramatically lessened or reversed when individuals move preferentially toward patches conferring higher fitness. Evolutionary ecologists have long modeled nonrandom dispersal, focusing primarily on its effects on population density over space, the maintenance of genetic variation, and reproductive isolation. Here, we build upon previous work by calculating how the extent of local adaptation and the migration load are affected when individuals differ in their dispersal rate in a genotype-dependent manner that alters their match to their environment. Examining a one-locus, two-patch model, we show that local adaptation occurs through a combination of natural selection and adaptive dispersal. For a substantial portion of parameter space, adaptive dispersal can be the predominant force generating local adaptation. Furthermore, genetic load may be largely averted with adaptive dispersal whenever individuals move before selective deaths occur. Thus, to understand the mechanisms driving local adaptation, biologists must account for the extent and nature of nonrandom, genotype-dependent dispersal, and the potential for adaptation via spatial sorting of genotypes.

## Introduction

The spectacular match displayed between organisms and their environments – from freeze tolerance in Antarctic toothfish (*Dissostichus mawsoni*) to extreme thermotolerance in “Pompeii worms” (*Alvinella pompejana*) – reflects the cumulative action of natural selection over evolutionary time. Despite the importance of natural selection in generating adaptation to the environment (Harvey and Pagel 1991; Rose and Lauder 1996; Orzack and Sober 2001), other evolutionary processes, in particular nonrandom dispersal (Edelaar and Bolnick 2012) but also nonrandom mutation (Cairns et al. 1988), potentially contribute to the adaptive process.

**Figure 1:**
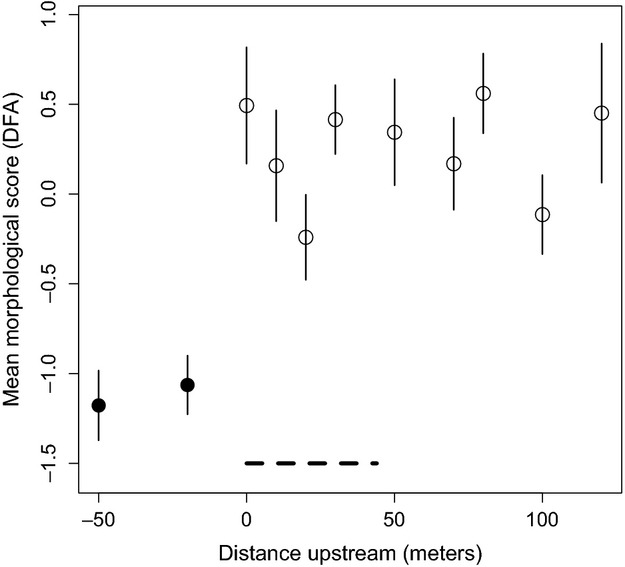
Phenotypic divergence between lake (solid circles) and inlet stream (hollow circles) threespine stickleback (*Gasterosteus aculeatus*) as a function of distance to the inlet (in meters). Points represent the mean discriminant function score (with ±1 SE bars) based on 23 morphometric landmarks of fish sampled at each location [17]. The phenotypic transition from lake to fully stream-like phenotypes is abrupt, occurring over a scale of meters. Individuals readily disperse this distance within days: the median dispersal distance over 4 days was 44 m (indicated by dashed horizontal line), with a maximum distance of over 150 m. Given this high migration rate, traditional migration-selection balance models would require unreasonably strong divergent selection, inconsistent with evidence of weak selection on reciprocal lake/stream transplants [4, 18]). Instead, the abrupt cline is likely maintained by phenotype-dependent habitat choice [17].

Evidence is accumulating that nonrandom dispersal must account for some of the observed match between organisms and their environment. In particular, some highly mobile organisms exhibit surprisingly abrupt genetic clines over small spatial scales without physical barriers to movement (Bolnick et al. 2009; Urban 2010; Richter-Boix et al. 2013). Often, biologists explain adaptive clines in terms of selection overwhelming the homogenizing effects of dispersal. However, when the typical dispersal distance is high relative to the cline width, one must invoke selection that is so strong as to be unrealistic in many cases. An alternative and more likely explanation is that dispersal is biased, with individuals moving in a manner that exaggerates the cline. For instance, in threespine stickleback (*Gasterosteus aculeatus*) individuals who morphologically resemble lake (stream) inhabitants are more likely to disperse into a lake (stream) site, maintaining strong morphological clines across just a few meters (Fig. 1). Furthermore, plant-feeding insects often exhibit biased oviposition in favor of plants on which their offspring have enhanced performance (Gripenberg et al. 2010), a pattern of nonrandom dispersal that contributes to the observed degree of local adaptation as well as the extent of reproductive isolation among host races (De Meeûs et al. 1995; Dres and Mallet 2002).

Despite clear evidence for nonrandom, genotype-dependent dispersal in nature (Edelaar et al. 2008) and theory illustrating its effects on adaptation and speciation (Armsworth and Roughgarden 2005, 2008; Armsworth 2009), the majority of population and quantitative genetic theory continues to assume that gene flow is random with respect to genotype. Furthermore, empiricists studying local adaptation are much more likely to ascribe genetic differences among populations to selection than to nonrandom gene flow, even when both may occur. For example, in numerous published studies, evidence for adaptive divergence (e.g., local adaptation in reciprocal transplants, high Q_ST_/F_ST_ ratios, or “genomic islands” of high divergence) is used to infer that divergent selection has overcome the constraining effect of gene flow. As we illustrate in this article, such adaptation may instead reflect a synergistic combination of divergent selection and biased gene flow. Our goal is to quantify the relative contributions of these two processes – selection and genotype-dependent dispersal – to local adaptation through the analysis of a one-locus, two-patch model.

## Terminology

Our focus is on nonrandom, genotype-dependent dispersal, defined as variation among genotypes in the probability of movement from a natal patch to the patch in which reproduction occurs. When dispersal rates reflect fitness differences among the genotypes, the term “fitness-associated dispersal” (Hadany et al. 2004; Armsworth and Roughgarden 2008) is often used. Other terms in the literature include “habitat selection” (Fretwell and Lucas 1970), “habitat choice” (Ravigné et al. 2004, 2009; Armsworth 2009), “conditional movement” (Armsworth 2009), “directed movement” (Armsworth and Roughgarden 2005), and some forms of “niche construction” (Odling-Smee et al. 1996; Donohue 2003). The range of terminology reflects, in part, different mechanisms thought to be responsible for variation in dispersal rates. For example, individuals may leave their natal habitat if they (i) are stronger dispersers, (ii) are locally less fit (in relative or absolute terms), (iii) expect higher fitness elsewhere, or (iv) prefer another habitat (for reasons that may or may not depend on fitness). To avoid implying that a particular mechanism is involved, we use the term genotype-dependent dispersal (GDD) throughout this article to refer to genotypic variation in dispersal rates.

Moving from one patch to another may lead to an increase in the fitness of a particular genotype relative to another genotype. In this situation, the genotype that stands to benefit from dispersal may be more likely to move, relative to the other genotype. We call this form of GDD “adaptive dispersal” because it contributes to the appearance of local adaptation. Importantly, as defined, adaptive dispersal need not imply that absolute fitness increases after dispersal; indeed, the absolute fitness of a genotype may decline, despite undergoing adaptive dispersal, if there are fewer resources and/or stronger competition in the new patch, such that the absolute fitness of a genotype that tends to disperse declines even though its relative fitness rises. By contrast, we refer to GDD as “maladaptive dispersal” when those individuals that tend to disperse experience lower relative fitness at their destination, compared with other genotypes. Thus, we use adaptive (or maladapative) dispersal to refer specifically to whether GDD contributes to (or opposes) changes in gene frequency due to local adaptation, rather than a more generic reference to dispersal toward good (or bad) patches for all individuals. Whether GDD is adaptive or maladaptive (in the sense of contributing to local adaptation) is highly context-dependent, depending on the particular genotypes and habitats examined, as well as the direction of dispersal.

It is also worth clarifying our use of the words “dispersal,” “fitness,” and “local adaptation,” given their varied use in the literature. We use “dispersal” to refer to genetic migration (Bull et al. 1987), in which an individual's movement alters where it breeds. Migration can refer to both transient movement (e.g., the migratory routes of birds) and to permanent movement, so for clarity we use “dispersal” to refer to the latter.

We follow standard practice in defining a genotype's fitness as its expected lifetime reproductive success, incorporating survival, mating success, and fecundity. In the context of GDD, however, what is meant by “expected” is ambiguous. It can refer to the expected fitness assuming that the genotype remains in its natal patch, the expected fitness if the individual switches habitat, or the expected fitness accounting for the probability of settling in each habitat. To distinguish among these possibilities, we use “anticipated fitness” to refer to an individual's fitness within a given habitat (allowing no subsequent dispersal). We use “realized fitness” to refer to lifetime expected fitness accounting for dispersal behavior. We measure the strength of natural selection according to the realized fitness, reflecting the actual differences in survival, mating success, and fecundity that genotypes experience. We also note that both “anticipated fitness” and “realized fitness” depend on the competition experienced, which may be based on levels of competition in the recent past or projected levels after other individuals disperse (in the following, we hold patch sizes constant, which equalizes competition for simplicity).

Finally, we use local adaptation to refer to the difference in fitness between individuals born in a patch versus individuals transplanted to that patch from elsewhere (“local vs. foreign” criterion; Kawecki and Ebert 2004). For the symmetric model that we primarily consider, however, this is equivalent to the difference in fitness between individuals that are raised in their natal patch versus raised in a non-natal patch (“home vs. away” criterion; Kawecki and Ebert 2004).

## Previous Work

An extensive body of theory on GDD has focused on how the pattern of dispersal should evolve in the presence of spatial variation in fitness. Much of this literature is focused on the evolution of movement patterns when resources vary over space, assuming all individuals are equally fit in each location. A classic result in ecological theory is that individuals will distribute themselves across space such that their densities are proportional to the resources available, the “ideal free distribution” (IFD; Fretwell and Lucas 1970; Fretwell 1972; Whitam 1980). The IFD assumes that individuals are free to move, do so without reference to other individuals, and are fully able to assess the resources remaining in each patch. If the relative amount of resources available in each patch remains constant over generations, then dispersal rates are predicted to evolve toward zero (Hastings 1983; Holt 1985). With temporal variation, however, evolution favors dispersal patterns in a manner that often approaches IFD (Holt and Barfield 2001).

More generally, unless the cost of habitat choice is strong, selection should favor the evolution of cognitive or physiological abilities required to (i) evaluate habitats and (ii) choose to settle in whichever habitat confers the highest expected absolute fitness given one's phenotype (Rausher 1984; Ruxton and Rohani 1999; Fry 2003; Armsworth 2009; Ravigné et al. 2009). Numerous models bear out the intuitive expectation that fitness-enhancing dispersal can evolve (De Meeûs et al. 1993; Ravigné et al. 2004, 2009; Armsworth 2009), and empirical evidence suggests such adaptive habitat preferences are found in a wide array of species (Jaenike 1985; Jaenike and Holt 1991; Harris and Jones 1995; Ahnesjö and Forsman 2006; Nosil et al. 2006; Edelaar et al. 2008; Lin et al. 2008; Bolnick et al. 2009; Clobert et al. 2009). The underlying logic is simple: individuals who choose habitat patches at random (in proportion to availability) will have lower fitness than individuals who can selectively disperse to patches conferring higher-than-average value to them. Because the conditions required for the evolution of fitness-enhancing dispersal are well established, for the remainder of the article we take it for granted that genotypes may differ in their propensity to disperse and that these differences may be adaptive. We then focus on the question of how GDD, once established, subsequently affects the dynamics of local adaptation. In doing so, we assume that the behavioral or physiological capacity for GDD remains fixed over the short-time scales during which we measure local adaptation. This assumption might be met, for example, in a metapopulation that first evolves a capacity for GDD and subsequently colonizes a new landscape where that pre-existing GDD is used to facilitate adaptation to different habitats. The newly colonized subpopulation might be polymorphic if, for instance, it is derived from immigrants from different habitats within the metapopulation.

Besides theory on the evolution of dispersal rates, previous work on GDD has explored its influence on reproductive isolation, particularly in the context of host-race speciation in phytophagous insects (Dres and Mallet 2002; Fry 2003). In addition, a growing body of theory explores the maintenance of protected polymorphisms when selection varies over space and individuals disperse nonrandomly (Garcia-Dorado 1986; De Meeûs et al. 1993; Ravigné et al. 2004, 2009). The conditions maintaining a protected polymorphism with spatially varying selection predict when we would expect to see local adaptation, with local genotypes faring better than foreign genotypes. These models do not, however, quantify the amount of local adaptation expected, nor do they assess the relative roles of selection and GDD in determining the level of local adaptation.

In contrast, comparatively few theoretical models have examined the allele frequency dynamics and expected equilibrium resulting from the combined effects of GDD and selection (De Meeûs et al. 1993; Hadany et al. 2004; Armsworth and Roughgarden 2005, 2008; Armsworth 2009), which is our focus here. Armsworth and Roughgarden used numerical simulations to show that adaptive dispersal mitigates the deleterious effects of gene flow on local adaptation, thus promoting between-population divergence and causing steeper clines (Armsworth and Roughgarden 2005, 2008; Armsworth 2009). For instance, they considered a situation in which fitness varies between two discrete habitats (Armsworth and Roughgarden 2005) or continually across space (Armsworth and Roughgarden 2008), allowing individuals to disperse into adjacent habitat patches that provide higher fitness. This “fitness-dependent dispersal” led to more pronounced clines than was possible under migration-selection balance. Consequently, selection and dispersal – specifically, adaptive dispersal – can have synergistic rather than opposing effects. This insight was also provided by Edelaar et al. (2008), who presented a verbal model arguing that dispersal can play a constructive role in adaptive evolution by spatially sorting genotypes in a manner that improves fitness of individuals above their anticipated fitness.

Despite these previous studies, the idea that dispersal can facilitate rather than hinder adaptation to local environmental conditions has still not gained wide traction in current evolutionary research. New ideas take time to filter into common practice, but adaptive dispersal, especially adaptive habitat choice, is quite a venerable concept, especially as a mechanism contributing to reproductive isolation and speciation (Maynard Smith 1966). We suspect that the role of GDD remains under-appreciated because previous work has not quantified the relative contributions of selection and GDD toward local adaptation. Thus, it is difficult for empiricists to evaluate whether observed patterns of local adaptation can be explained primarily by selection (little or no GDD) or whether GDD plays a major role. To this purpose, we develop a simple analytical model that provides a quantitative statement about how genotype-dependent dispersal affects between-population divergence and mean fitness. Using a combination of analytical and numerical techniques, we quantify the importance of GDD to local adaptation and explore how GDD alters the migration load typically experienced when individuals disperse across a heterogeneous environment. Our results highlight the potentially large impact of GDD on adaptation and therefore the need to habitually consider nonrandom gene flow in evolutionary studies of adaptation to local conditions.

## Model

Our goal is to determine the extent to which GDD modifies the rate or extent of local adaptation, in comparison with the migration-selection balance predicted if dispersal rates were genotype-independent. We assume that the focal species has a pre-existing and genetically fixed capacity for GDD, which remains constant over the time period during which local adaptation is measured. We leave consideration of simultaneous evolution of dispersal behavior and adaptation for future work.

We constructed a one-locus haploid model with migration between two habitat types, within which different alleles are favored (*a* in habitat *A* and *b* in habitat *B*). Individuals are born in their parents' habitat, and the frequency of alleles *a* and *b* among the newborns in habitat *i* at generation *t* is *p*_*t*_(*a,i*) and *p*_*t*_(*b,i*), respectively. Each generation is divided into a dispersal phase and a selection phase, so individuals have the opportunity to disperse prior to the action of selection. At the time of selection, individuals have fitness of *w*(*a*,*A*) = 1 or *w*(*b*,*A*) = 1−*s*_*A*_ in habitat A and *w*(*a*,*B*) = 1−*s*_*B*_, or *w*(*b*,*B*) = 1 in habitat B. Because juveniles disperse before selection acts, their realized fitness may differ from their anticipated fitness (e.g., an individual bearing allele *a* born in patch *B* has an anticipated fitness of *w*(*a*,*B*) but if it disperses to patch A its realized fitness is *w*(*a*,*A*)). After juvenile dispersal and selection, we assume the adult population size reaches a constant value of *N*_*i*_ in habitat *i* (a fixed carrying capacity), with each habitat contributing a constant number of offspring to the next generation. Because we assume that neither reproduction nor population regulation change the allele frequency within a patch, their exact timing during the life cycle is irrelevant, as long as each patch contributes to the dispersing phase in the next generation in proportion to *N*_*i*_ and dispersal is followed by selection followed by a census. For example, the same recursion equations would apply if adults disperse, then experience selection, followed by reproduction, and then a census, with population regulation among either the surviving adults or the juveniles.

Although one might instead model selection and dispersal as simultaneous processes, the subdivision of each generation into two time steps makes it easier to calculate the relative effects of GDD and selection. The ordering of dispersal before selection is also expected if GDD has evolved to improve matching between an individual's phenotype and the environment, thereby lessening selection against individuals born into a mismatched environment. The reverse order might also be biologically relevant (especially in organisms, like plants, where dispersal occurs early in life); the impact of GDD on selection would then not be felt until the next generation.

Following a bout of dispersal, the frequency of allele *a* in habitat *i* changes to


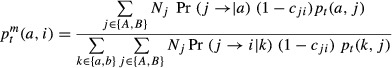
(1)

The term 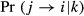 represents the probability that genotype 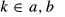 emigrates from natal habitat *j* to habitat *i*. With strictly random dispersal, this probability would be a genotype-independent constant (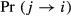), potentially reflecting the relative availability of the two habitat types or habitat preferences shared by all genotypes. With GDD, we instead allow each genotype to have different, but constant, dispersal probabilities. A cost of dispersal 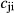 is included to reflect an increased probability of dying during dispersal relative to nondispersers 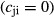.

Following dispersal, the allele frequency in each habitat is further altered by selection, which depends on the realized fitness of each individual. The resulting allele frequency in patch *i* at the start of the next generation is:


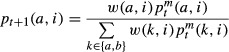
(2)

Overall, this model extends a classic model of maintenance of genetic variation (Levene 1953; Christiansen 1975) to the case of nonrandom dispersal.

We can relate the parameters of this model to the degree of local adaptation observed at time *t*. Specifically, using the “local vs. foreign” criterion, the degree of local adaptation within patch A equals:


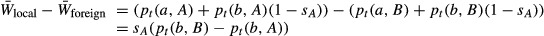
(3a)

while the degree of local adaptation using the “home-vs.-away” criterion for individuals sampled from patch A equals:


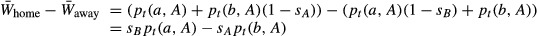
(3b)

(Kawecki and Ebert 2004). The corresponding measures in patch B can be obtained by interchanging A and B as well as *a* and *b*. Here, we assume that local adaptation is measured in such a way that transplanted individuals must remain where placed (i.e., subsequent dispersal is prevented), consistent with typical experimental methods.

The dynamics of dispersal described by equation (1) is general, allowing for any possible mechanism of GDD. We use this general formulation to explore the consequences of GDD for local adaptation. Importantly, equation (1) allows for migration rates that are correlated or uncorrelated with the fitness of an individual. Indeed, each genotype could have equal fitness and yet GDD could generate allele frequency divergence between patches.

To supplement this general analysis of local adaptation, we also consider a specific form of GDD involving habitat choice, where individuals evaluate their likely fitness in each patch. Here, we assume that some or all individuals make forays to other habitats and evaluate their anticipated fitness at each site (or some fitness correlate). For instance, individuals might detect different levels of food accessibility, energetic stress due to locomotion demands, temperature stress, or predation risk. Before these fitness differences are realized as variation in survival or fecundity, the individuals choose to disperse or remain in their natal patch for breeding, preferring whichever habitat confers higher anticipated fitness. Proximally, this choice may be mediated by, for example, relative levels of stress hormones or energetic income experienced in each patch. For the sake of generality, we do not attempt a mechanistic model specifying the physiological or neurological basis of habitat choice decisions. Rather, we describe the process phenomenologically, assuming that the dispersal probability of an individual from patch *j* to patch *i* depends on that individual's anticipated fitness in patch *i* relative to its average anticipated fitness across the two patches:


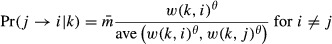
(4)

where *θ* determines the direction and strength of habitat choice and ave() is the arithmetic average. When *θ* = 0, all individuals migrate at equal rate (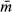, random dispersal, no GDD), while *θ* > 0 corresponds to adaptive dispersal (Fig. S1) and *θ* < 0 to maladaptive dispersal. The function (eq. 4) is mathematically equivalent to one used in foraging theory to represent the probability of switching from a suboptimal to an optimal resource, depending on the relative values of the resources and forager sensitivity (Schreiber et al. 2006; Schreiber and Lloyd-Smith 2009). In addition, we explored dispersal rates that were a linear function of an individual's perceived fitness in different patches, 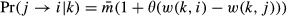, or a function of an individual's anticipated fitness compared with another genotype (*l*) in the natal patch, 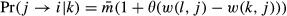. The results were qualitatively similar (see Supplementary *Mathematica* file Data S1).

An important limitation of both the general dispersal model (eq. 1) and the specific examples described above is that the dispersal rate of each genotype does not depend on the genotype frequencies within each patch. Density-dependent dispersal is also ignored (population densities are assumed large and constant, ignoring genetic drift). We acknowledge that local densities and genotypes may reduce patch quality and affect dispersal decisions. However, local adaptation can also be driven by abiotic conditions, by resource traits (e.g., defensive compounds, as opposed to resource quantity), or by natural enemies whose abundance need not be regulated by the density of the focal organism. Frequency- or density dependence could be explicitly incorporated into the dispersal terms, 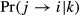, but we do not do so here. We also assume that there is no de novo mutation. Obviously, any application to a specific empirical system would benefit by including a mechanistic model of patch sampling and decision-making regarding dispersal and any frequency- or density dependence as relevant. Our goal, however, is to use a plausible basic model to assess the extent to which GDD alters the speed and total amount of local adaptation.

To obtain more analytically tractable results, we primarily restrict our attention to a symmetrical version of the model, where patches are equally productive (*N*_1_ = *N*_2_) and where both genotypes gain similar fitness benefits in their optimal habitats and exhibit similar dispersal behaviors. While the symmetry assumption reduces the generality of our analysis, it greatly simplifies the dynamics and yields more readily interpretable results. Furthermore, the results presented are similar to those obtained from the more general asymmetrical model (both the exact equilibrium and numerical code to analyze the dynamics are presented in the Supplementary *Mathematica* file Data S1). In supplementary figures, we illustrate how breaking symmetry affects the results presented in the main text.

In the context of Ravigné et al.'s (2009) classification scheme for habitat choice models, the model examined here includes local regulation and constant habitat output (no variation in density dependence), arbitrary local adaptation trade-offs (their category 1), fixed strength of habitat choice, and a combination of philopatry and habitat choice, which may or may not be adaptive. The main point of departure from the classification scheme of Ravigné et al. is that our focus is not on when a polymorphism is maintained, but rather the extent of differentiation across habitats and the relative contribution of GDD versus selection in determining the extent of local adaptation. To this end, we used deterministic numerical simulations (implemented independently by the two authors in R and *Mathematica*, respectively) and analytical proofs to evaluate how the strength of GDD, strength of selection, and mean dispersal rate influence the rate and extent of adaptation and divergence between populations.

## Results

### Analytical results for a symmetrical model

We first derive an analytical solution for the general model in the perfectly symmetrical case where selection acts equally against the locally disfavored allele in each habitat (*s*_*A*_ = *s*_*B*_ = *s*), individuals of a given genotype dispersal with equal probability, the cost of dispersal is the same for all dispersing individuals (*c*), and patches are of equal size (*N*_*A*_ = *N*_*B*_). The latter assumption equalizes the contribution of offspring from each patch to the next generation, which is known to facilitate the maintenance of balanced polymorphisms (Levene 1953; Maynard Smith 1966; Bulmer 1971; Ravigné et al. 2009). Asymmetric selection or migration does not substantially change the qualitative results, except that polymorphism is less likely to be maintained and the equilibria is more cumbersome, being the root to a quadratic whose terms are lengthy functions of the parameters (see Supplementary *Mathematica* file (Data S1) for more detailed results from both the symmetric and asymmetric model). For clarity, we replace the general dispersal functions (eq. 1) with:


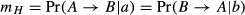
(5a)

and


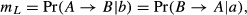
(5b)

which represent the dispersal probability of individuals that have a high or low match to their natal habitat in the symmetrical case (e.g., *m*_L_ applies to poorly matching individuals with allele *a* in habitat *B* or allele *b* in habitat *A*). This formulation is agnostic about the mechanism by which individuals choose a habitat (e.g., habitat preference, levels of stress hormones, detecting resource availability, etc.).

We measure the strength of GDD as 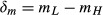. Our goal is to compare the degree of local adaptation when alleles affect dispersal rates 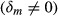 to the case where the alleles have no effect on dispersal 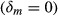. To minimize differences between the strategies being compared, we hold the average of the two migration rates, 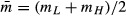, constant. This choice has the benefit that 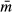 does not vary as the population evolves. Note, however, that 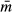 is not the average dispersal rate that would be empirically observed in a population, which would depend on the allele frequencies in each patch. Adaptive dispersal corresponds to cases of GDD where individuals migrate at a higher rate from locations where they have lower potential fitness 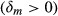, with maladaptive dispersal occurring when 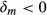


Assuming that the system has reached a point where the high-fitness allele (*a* in *A* and *b* in *B*) is at the same frequency in both patches (*p*_*t*_ = *p*_*t*_(*a,A*) = *p*_*t*_(*b,B*)), the high-fitness allele will thereafter remain at equal frequencies in both patches, and its dynamics are governed by the recursion equation:


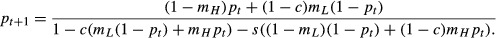
(6a)

In this symmetrical model, the degree of local adaptation defined using either the “local vs. foreign” criterion (3a) or the “home-vs.-away” criterion (3b) simplifies to 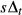, where 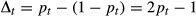 represents the degree of genetic differentiation between the patches (the difference in frequency of an allele where it is favored and where it is disfavored). The degree of local adaptation thus rises linearly with the frequency of the fit allele in each patch, *p*_*t*_.

To simplify the presentation below, we group terms in equation (6a) involving *p*_*t*_:


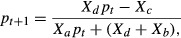
(6b)

With


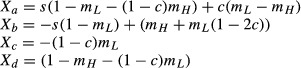


When dispersal is present, there is a single valid equilibrium point 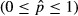 of this symmetric model at:


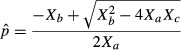
(7)

For the population to exhibit local adaptation 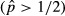 requires that dispersal is either adaptive or only weakly maladaptive:


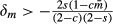
(8)

(detailed proofs are available in the Supplementary *Mathematica* package Data S1). We further confirmed the stability of equilibrium (eq. 7) in the full dynamical system (with *p*_*t*_(*a,A*) and *p*_*t*_(*b,B*) allowed to differ), as detailed in the Supplementary *Mathematica* package (Data S1). If condition (eq. 8) is satisfied, then equilibrium (eq. 7) is locally stable (assuming 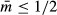). When condition (eq. 8) is not satisfied, however, then either the population fixes on one of the alleles or it approaches an equilibrium exhibiting local maladaptation 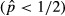, depending on the parameters. We assume in what follows that local adaptation is observed (condition (8) is met so that 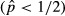) and focus on describing the extent of the local adaptation caused by GDD.

**Figure 2:**
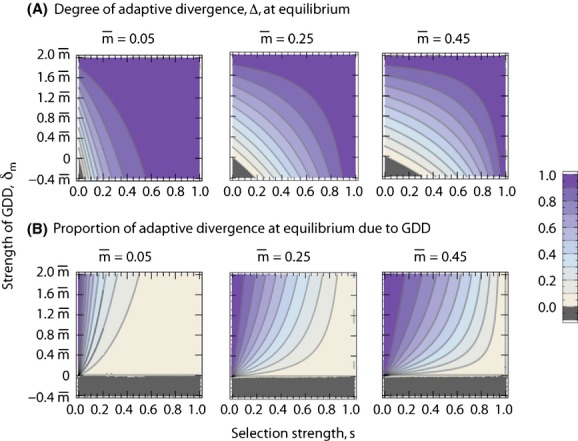
Local adaptation with genotype-dependent dispersal at equilibrium. (A) Top panels show the extent of population divergence in allele frequency at equilibrium, 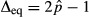, as the extent of GDD is varied (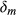, vertical axis) for a given strength of selection (horizontal axis) and average migration rate (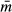, increasing in panels from left to right). With maladaptive dispersal (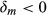), local adaptation cannot be maintained in the gray regions because maladaptive dispersal overwhelms selection (eq. 8 is not met). (B) Bottom panels show the proportion of local adaptation at equilibrium that can be attributed to GDD (eq. 9). In each panel, the maximum degree of GDD is set such that individuals that match their habitat do not move (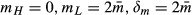). Here, we assume selection and migration are symmetric in the two habitats. Similar trends hold when selection or migration strengths are asymmetric (Fig. S4), as long as both alleles are present. Similar trends also hold with fitness-dependent dispersal (eq. 4; Fig. S2) and with costs of dispersal (Fig. S3).

At equilibrium, we define the proportion of local adaptation attributable to GDD as:


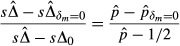
(9)

where the numerator represents the difference in the degree of local adaptation with GDD 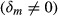 and without 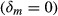 and the denominator represents the total amount of local adaptation observed relative to the case where the allele frequencies are equal in both patches (no local adaptation; i.e., 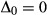 at 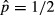). The equilibrium level of genetic differentiation attributable to GDD (eq, 4), Fig. S3 shows the same result with dispersal costs, and Fig. S4 shows the effects of having either asymmetric selection or migration. All four figures show that GDD can account for the majority of observed adaptive divergence. Specifically, the magnitude of allele frequency divergence and local adaptation can be more than twice as large with GDD than without, across a substantial portion of the parameter space. Interestingly, GDD is especially effective at amplifying adaptive divergence when migration rates are high or selection weak. High migration rates allow for greater differences in dispersal among genotypes, increasing the potential for GDD to impact local adaptation (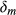 is at most 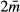, so that the scope for GDD increases with the average migration rate). When potential selection (*s*) is weak, selection is readily overwhelmed by random gene flow, so that little local adaptation is observed unless GDD is present (Fig. 2B). In this case, even mild levels of GDD can substantially augment equilibrium divergence. If the extent of GDD depends on the strength of selection, as is true with the specific model of habitat choice (eq. 4), then dispersal differences will become weaker as selection becomes weaker (Fig. S1); even then, GDD continues to contribute most of the observed degree of local adaptation when selection is weak and migration frequent (Fig. S2B). Interestingly, costs of migration, even strong costs (*c* = 0.5), have relatively minor effects on the proportion of local adaptation attributable to GDD (compare Figs 2 and S3). However, these costs might in the long run select against GDD or dispersal in general.

Next, we ask how GDD affects the extent of local adaptation at equilibrium. For a given average dispersal rate (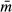), increasing the amount of adaptive dispersal always increases the extent of local adaptation, 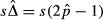, because


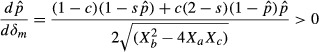
(10)

(all parenthetical terms are positive, see Supplementary *Mathematica* file Data S1). This result proves, in general, that we expect to see increased local adaptation at equilibrium given more adaptive dispersal. Similarly, as one might expect, increasing the strength of selection increases the degree of local adaptation 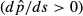, while increasing the average amount of migration has the opposite effect 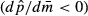, as detailed in the Supplementary *Mathematica* file (Data S1).

Finally, we can iterate the recursion equation to obtain a general solution for the locally favored allele frequency at any future point in time:


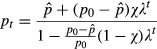
(11)

where


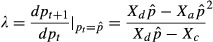



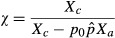


The 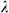 term equals the eigenvalue of the symmetric system evaluated at the equilibrium with *p*(*a,A*) = *p*(*b,B*) and always lies between −1 and +1 (Sup. *Mathematica* file). The approach of the system to equilibrium can be oscillatory, with the system overshooting the equilibrium (evaluated after selection in each generation), if 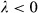, which requires that:


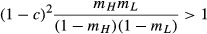
(12)

and only occurs when the migration rate is very high (*m*_*L*_ and/or *m*_*H*_ >1/2). The 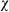 term alters the shape of the approach to equilibrium. When 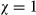, the system approaches its equilibrium geometrically by a factor 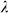 each generation.

**Figure 3:**
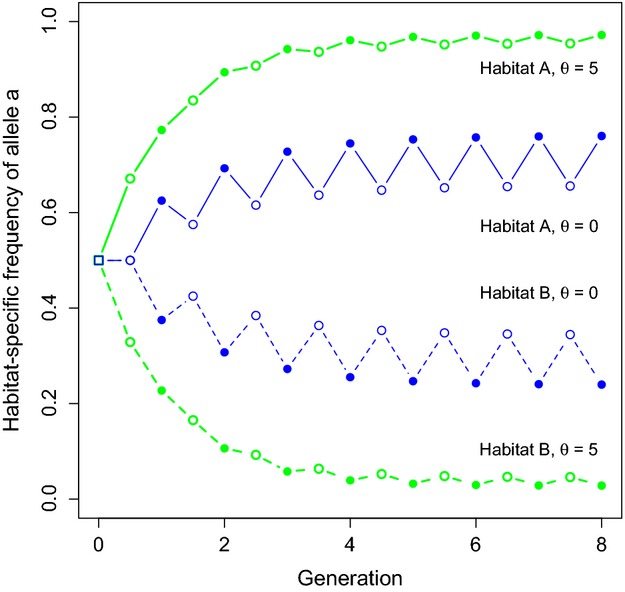
Genotype-dependent dispersal hastens allele frequency divergence between habitats. Lines present the frequency of allele *a* in habitat *A* (solid lines) and *B* (dashed lines) with GDD (green) and without GDD (blue). Each generation consists of two time steps, corresponding to dispersal (open circles) followed by selection (filled circles). Without GDD, dispersal always reduces the divergence between populations, as indicated by the convergence of the blue open circles. With GDD, however, divergence can increase even during the dispersal phase, especially during the early time points (green open circles). Parameters: *s*_*A*_ = *s*_*B*_ = 0.4, 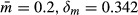 (equivalent to θ = 5 under the specific model of habitat choice, eq. (4). Similar results are seen for other initial allele frequencies (Fig. S7).

Figure 3 illustrates the dynamics of local adaptation with GDD. In the first generation of the simulation, migration with GDD leads to substantial divergence in allele frequency between habitats and thus increased mean fitness and local adaptation. Notably, this divergence occurs before the first generation is subjected to any differential mortality (realized selection). As a result, at the end of the first generation, more than half of the observed differentiation in allele frequencies, 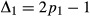, is due to GDD (green vs. blue curves).

Again, the fraction of adaptation attributable to GDD at time *t* can be defined as:


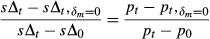
13

For example, starting with equal allele frequencies everywhere (*p*_0_ = 1/2) and no local adaptation, the amount of local adaptation caused by GDD after one generation of dispersal and selection (*t* = 1) is:


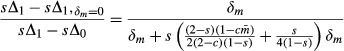
(14)

(Fig. S5). Equation (14) is always positive when 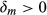, indicating that adaptive dispersal always hastens the initial build up of local adaptation, as expected. When selection is weak (small *s*), equation (14) approaches one, demonstrating that GDD can drive most of the observed increase in local adaptation. Indeed, selection can be absent (*s* = 0), and yet GDD will drive differentiation of the populations, with genotype *a* (*b*) becoming more common in patch A (B) when 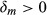. This pattern might generate the appearance of adaptive divergence (e.g., a repeatable phenotype-environment correlation), despite the fact that selection is not involved and there is no local adaptation (immigrants are not less fit).

The general solution allows us to solve for the time *t* until the system traverses a proportion, α, of the distance between the starting point and the equilibrium degree of local adaptation, by solving 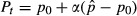 for *t*:


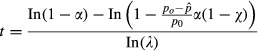
(15)

Fig. S6 shows that the time to reach equilibrium generally decreases slightly with GDD when 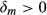, indicating that adaptive dispersal both permits a higher degree of local adaptation at equilibrium and hastens the approach to this equilibrium. Again costs of migration have relatively minor effect (Fig. S6B).

Our results make it clear that one can observe a given divergence between two populations with or without GDD. There are several unique features of GDD, however. First, GDD can explain fine-scale adaptive divergence in highly mobile organisms even when fitness differences are minor (e.g., Fig. 1). Second, GDD allows a faster approach to a given equilibrium divergence than is possible with selection and genotype-independent dispersal. Third, because the dispersal rate of individuals with GDD depends on the genotypes present in each patch, the total dispersal rate changes dynamically over time as genotype frequencies change (Figs. S7, S8), a phenomenon not typically considered in migration-selection balance models. When a majority of individuals within a population are poorly adapted, adaptive dispersal leads to a higher emigration rate than would be expected given random movement at rate 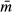. Once populations become locally adapted, individuals remain in their habitat more often with adaptive dispersal than without. This reduction in gene flow can contribute to reproductive isolation between populations in different habitats, an effect often invoked in the context of insect host-race speciation (De Meeûs et al. 1995; Dres and Mallet 2002; Fry 2003). A fourth distinguishing feature of GDD is that the individuals that disperse are a biased sample of the genotypes within a population (Fig. S9).

### The impact of GDD on genetic load

**Figure 4:**
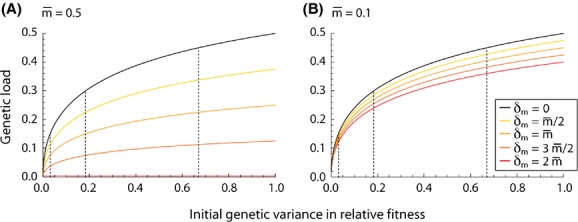
The relationship between variance in fitness at the start of a generation and the resulting genetic load, for two mean migration rates (A, B). Genetic load is defined as the reduction in mean fitness below its maximum of one, measured here in the first generation starting with no local adaptation (*p*_0_ = 0.5). For a given strength of selection, genetic load is mitigated by adaptive dispersal (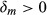) relative to the case when migration is random (black curve; 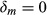). This result occurs because individuals move out of habitats where they are less fit, prior to experiencing the expected mortality. GDD is most effective at reducing genetic load when low fitness individuals are much more likely to disperse than high-fitness individuals (large 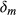). Observe that complete adaptive dispersal (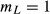 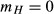, so that 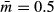 and 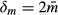) eliminates the load entirely (red curve in panel A lies along the horizontal axis). Variance in fitness is measured before migration or selection, and load is measured during selection, after migration. Parameters: (A) 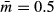, (B) 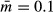, with *s* varying from 0 to 1 (the three dashed vertical lines correspond to *s* = 0.3, 0.6, and 0.9, from left to right). See Fig. S10 for the same result with different initial allele frequencies.

Perhaps most importantly, GDD translates anticipated variation in fitness into migration over space, rather than mortality or failure to reproduce (“genetic load”). As a result, GDD entails less genetic load to achieve a given amount of local adaptation (Fig. 4). Indeed, when GDD is strong (Fig. 4A), substantial adaptive divergence can occur despite negligible genetic load because individuals emigrate to avoid areas of low anticipated fitness. As an extreme case, if habitat preference is perfect then all individuals disperse to their optimal habitat leaving no variance in realized fitness and no load (red curve in Fig. 4A). Therefore, adaptation can in principle proceed without realized selection (though not without anticipated selection, hence the need to distinguish the two definitions of selection). The load-mitigating effect of GDD is most pronounced when dispersal capacity is high (compare Fig. 4A and B), but even for weak dispersal (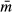 = 0.1, Fig. 4B) strong GDD can reduce the genetic load due to strong selection (*s* ∼ 1) by 20%.

## Discussion

It is well known that spatially varying selection can favor the evolution of adaptive dispersal (Rausher 1984; Ravigné et al. 2009) and that habitat preferences vary among conspecific individuals within many species (Jaenike and Holt 1991; Edelaar et al. 2008). Previous verbal and simulation models suggested that once adaptive dispersal has evolved, it can lead to more substantial local adaptation than is possible when selection is opposed by random migration (Armsworth and Roughgarden 2005, 2008; Edelaar et al. 2008; Armsworth 2009). Here, using a simplified two-allele, two-patch model, we quantify the extent to which genotype-dependent dispersal amplifies and accelerates local adaptation. The novel contribution of the current work is that we have explicitly partitioned the degree of local adaptation due to GDD (spatial sorting of alleles) versus selection (realized variation in mortality or fecundity). We show that in some situations GDD may be responsible for the majority of divergence observed between neighboring populations.

GDD most strongly increases adaptation when the potential for movement is quite high (right panels, Fig. 2A and B). Therefore, GDD will be most important in situations where environmental gradients are steep relative to individuals' dispersal ability (Armsworth and Roughgarden 2008; Armsworth 2009), such as fine-grained habitat mosaics or abrupt clines (e.g., Fig. 1). In contrast, natural selection is most effective at driving divergence when dispersal is rare. Thus, natural selection and GDD are most effective at driving adaptation at different ends of a continuum of dispersal abilities. This insight may help guide empiricists to focus on GDD or selection as likely primary causes of adaptation, depending on the spatial scale of dispersal and divergent selection.

While we have focused our attention on adaptive dispersal, maladaptive dispersal is also possible, especially if only high-fitness individuals have sufficient resources to disperse successfully (Bowler and Benton 2005; Bonte and de al Pena 2009). This model and our results readily extend to this case (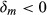). Not surprisingly, maladaptive dispersal hinders the effect of natural selection and can even prevent local adaptation when it would have otherwise been feasible (eq. 8; Fig. 2). Consequently, species with positive condition-dependent dispersal or other mechanisms of maladaptive dispersal will be less likely to exhibit local adaptation or undergo spatial divergence leading to speciation.

Several caveats are worth noting. First, we do not examine the dynamics of how GDD or dispersal rates evolve concurrently with the process of adaptation. Other studies have modeled the evolution of GDD (Rausher 1984; Rice 1984; Ravigné et al. 2004, 2009; Armsworth 2009), but we assumed that GDD remains relatively fixed over the period of time during which local adaptation is being measured. Second, we consider only a simplified two-patch habitat model without explicit spatial structure. More complex environmental landscapes might lower the ability of individuals to disperse adaptively (e.g., if two habitats are large and parapatric, such that GDD is only available to individuals near the habitat border). Finally, we do not consider feedback between adaptation and population size, with the resulting complications arising from density-dependent fitness and asymmetric migration loads. Future analyses could profitably evaluate the effects of relaxing these various assumptions.

## Implications

GDD has several major implications. First, we cannot assume that natural selection (realized variance in fecundity or survival) is always the primary cause of adaptive divergence between adjoining populations. Instead, the specter of future low fitness may induce individuals to disperse to higher-fitness locations, thereby evading selection and generating substantial divergence over small spatial scales. As a result, loci affecting dispersal would exhibit genetic differentiation among populations (higher *F*_ST_ than the genomic background). At present, when genome scans reveal peaks of *F*_ST_, authors interpret the pattern as an effect of selection (Stinchcombe and Hoekstra 2007; Nosil et al. 2009). We argue that similar patterns could arise when divergence is primarily caused by habitat choice. Second, GDD can generate divergence over smaller spatial scales than we would expect under migration-selection balance with random movement. Third, adaptive GDD can facilitate geographic range expansion by reducing the swamping effects of gene flow into peripheral populations that has been proposed to constrain species' geographic ranges (Kirkpatrick and Barton 1997). Fourth, conservation biologists may need to consider how GDD affects the adaptability of populations of mobile animals in heterogeneous landscapes. For example, in species where GDD is adaptive, habitat connectivity may be essential to allow adaptation to changing environments, whereas the same connectivity may be detrimental to randomly dispersing organisms that suffer migration load. Fifth, GDD facilitates adaptation without genetic deaths (Fig. 4). This relaxed genetic load could maintain a higher effective population size (and weaker genetic drift) than is possible with natural selection alone. Finally, once local adaptation is established, strong adaptive dispersal can substantially reduce gene flow between habitats, thereby promoting speciation via habitat-based reproductive isolation (Rice 1984; Rice and Salt 1988; Diehl and Bush 1989; De Meeûs et al. 1993; Fry 2003; Eroukhmanoff et al. 2011). Adaptive dispersal thus acts as a so-called speciation-facilitating “magic trait” (Gavrilets 2004; Servedio et al. 2011).

In light of the potentially major effects of GDD, we believe that biologists studying adaptation in nature should habitually test for GDD, especially when the spatial scale of divergence is small relative to the mobility of the organism. There are a variety of ways to test for nonrandom gene flow (Edelaar et al. 2008; Edelaar and Bolnick 2012), for instance by comparing the genotypes of dispersers to residents, or by tracking dispersal behavior of individuals after experimental manipulations of either their phenotype or their environment. Ideally, one could simultaneously conduct classical reciprocal transplant experiments to test for divergent natural selection (constraining individual dispersal), supplemented with reciprocal transplants in which individuals are free to disperse to their optimal habitat. Comparisons of the rate and magnitude of adaptive change in constrained (selection only) and unconstrained (selection and GDD) experiments could effectively reveal the relative effects of these adaptive processes. Such experiments would need to control for mere philopatry, such as arises when individuals imprint on their natal environment.

Dispersal is not necessarily the random process typically assumed by evolutionary biologists (Edelaar and Bolnick 2012). Our analysis shows that nonrandom gene flow, in the form of genotype-dependent dispersal, can substantially facilitate adaptation. Consequently, adaptation can arise with surprisingly little realized variation in survival or fecundity. Although GDD has been demonstrated in nature, it remains to be seen how pervasive GDD really is. However, this model makes clear that GDD may be more influential than is widely appreciated and therefore should be more routinely considered by theoreticians and experimentalists alike.

## Supporting Information

**Figure S1.** Probability of dispersal in the specific model of habitat choice (eq. 4).

**Figure S2.** Local adaptation with genotype-dependent dispersal at equilibrium.

**Figure S3.** Local adaptation at equilibrium with genotype-dependent dispersal when migration is costly.

**Figure S4.** Degree of population divergence in allele frequency, Δ_eq_, with asymmetric selection (A) or migration (B) in the two habitats.

**Figure S5.** Amount of first-generation divergence in allele frequency due to GDD.

**Figure S6.** Time to approach equilibrium. Shown is the number of generations required for the system to move 95% of the way to equilibrium (α = 0.95).

**Figure S7.** Adaptive dispersal increases allele frequency divergence between habitats.

**Figure S8.** The effective migration rate between habitats changes dynamically with GDD.

**Figure S9.** Genotype-dependent dispersal causes emigrating individuals to be a biased sample of the individuals within a habitat.

**Figure S10.** The relationship between variance in fitness at the start of a generation and the resulting genetic load, for two mean migration rates (A, B).

**Data S1.** Data sufficient to recreate the empirical plot of divergence between lake and stream stickleback, Fig. 1. The data were collected and analyzed as originally described in Bolnick et al. (2009). Evolution. The data had not previously been presented in the same form as in Fig. 1.
