# Supplementary material for: The magnitude of local adaptation under genotype-dependent dispersal
Source: Ecol Evol. 2013 Oct 30;3(14):4722–35. doi: 10.1002/ece3.850 (PMC3867907; doi:10.1002/ece3.850)
Supplement: Supplementary file 1 [file ece30003-4722-SD1.docx]

**Supplementary Information**


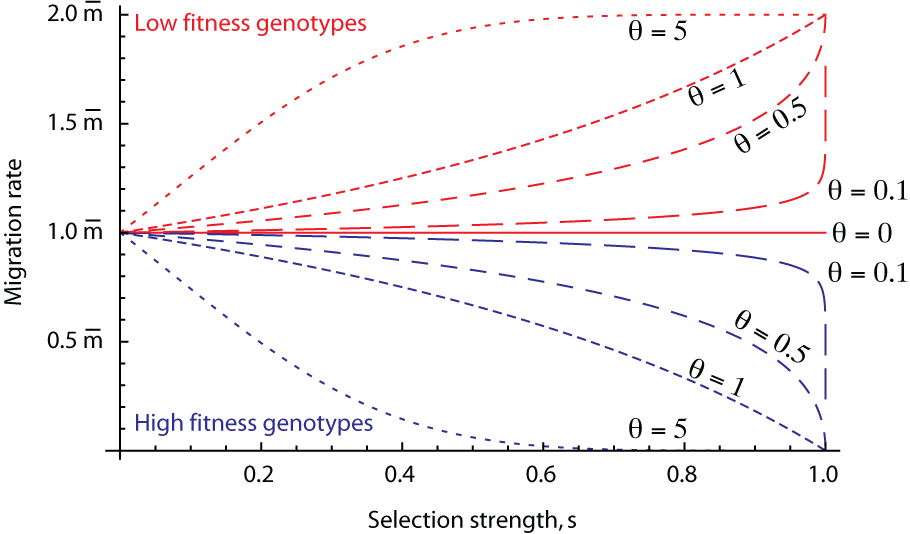


**Figure S1.** Probability of dispersal in the specific model of habitat choice (eq. 4). The figure shows the dispersal rate for individuals that have low anticipated fitness in their current environment (top red curves) versus high anticipated fitness (bottom blue curves) when dispersal is adaptive ($\theta$ > 0).


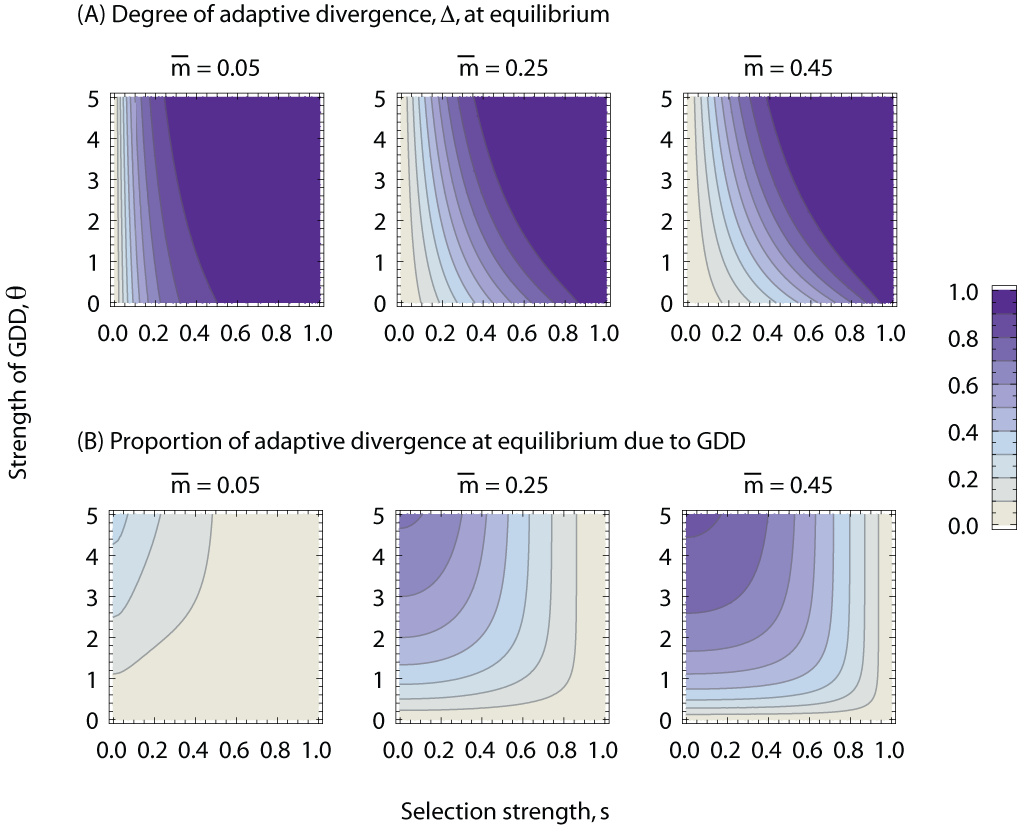


**Figure S2.** Local adaptation with genotype-dependent dispersal at equilibrium. Figure is equivalent to Figure 2, except using the specific model of adaptive habitat choice (4) with $\theta>0$. (A) Top panels show the extent of population divergence in allele frequency at equilibrium, $\Delta_{eq}$, as the extent of adaptive habitat choice is increased ($\theta$, vertical axis) for a given strength of selection (horizontal axis) and average migration rate (, increasing in panels from left to right). (B) Bottom panels show the proportion of local adaptation at equilibrium that can be attributed to GDD (equation 9).


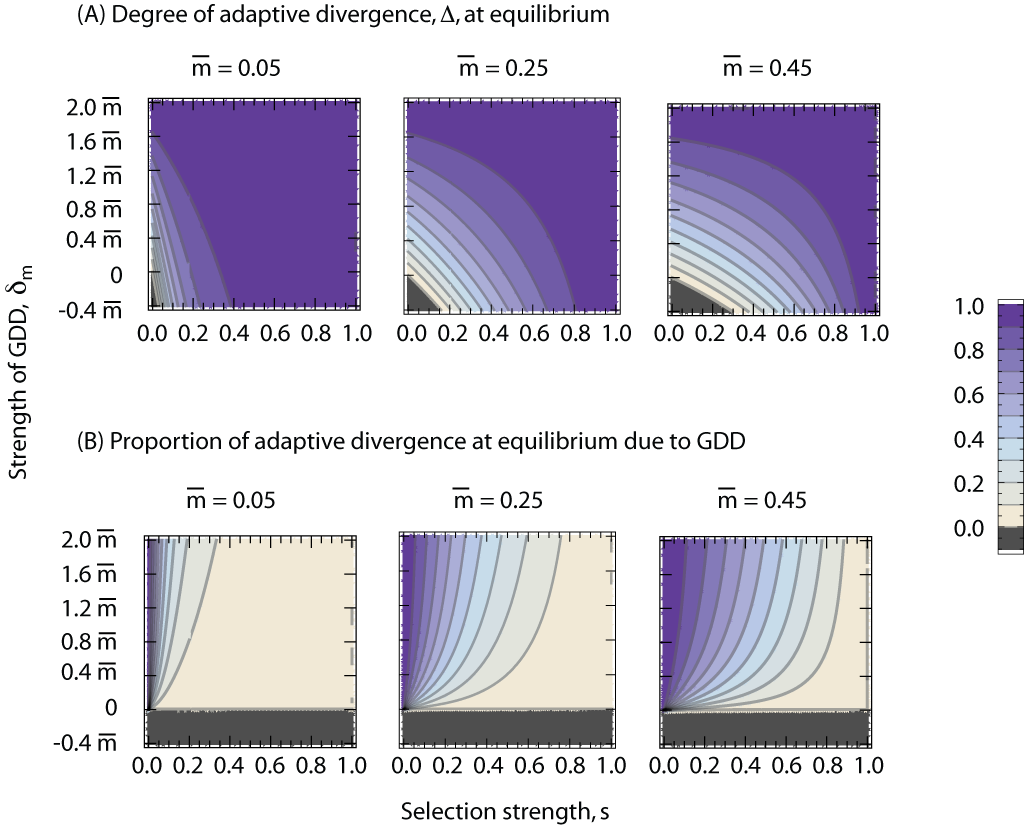


**Figure S3.**  Local adaptation at equilibrium with genotype-dependent dispersal when migration is costly. Fig. S3 is identical to Fig. 2, except with a 50% cost of migration (*c* = 0.5). (A) Top panels show the extent of population divergence in allele frequency at equilibrium, $\Delta_{eq}$, as the extent of adaptive habitat choice is increased ($\delta_{m}$, vertical axis). Costs of migration slightly increase the degree of adaptive divergence (compare to Fig. 2A) by reducing the effective migration rate. (B) Bottom panels show the proportion of the adaptive divergence at equilibrium that can be attributed to GDD (equation 9). Costs of migration slightly decrease the role of GDD because of the lower effective migration rate, but the magnitude of the effect of costs is very small (compare to Fig. 2B).


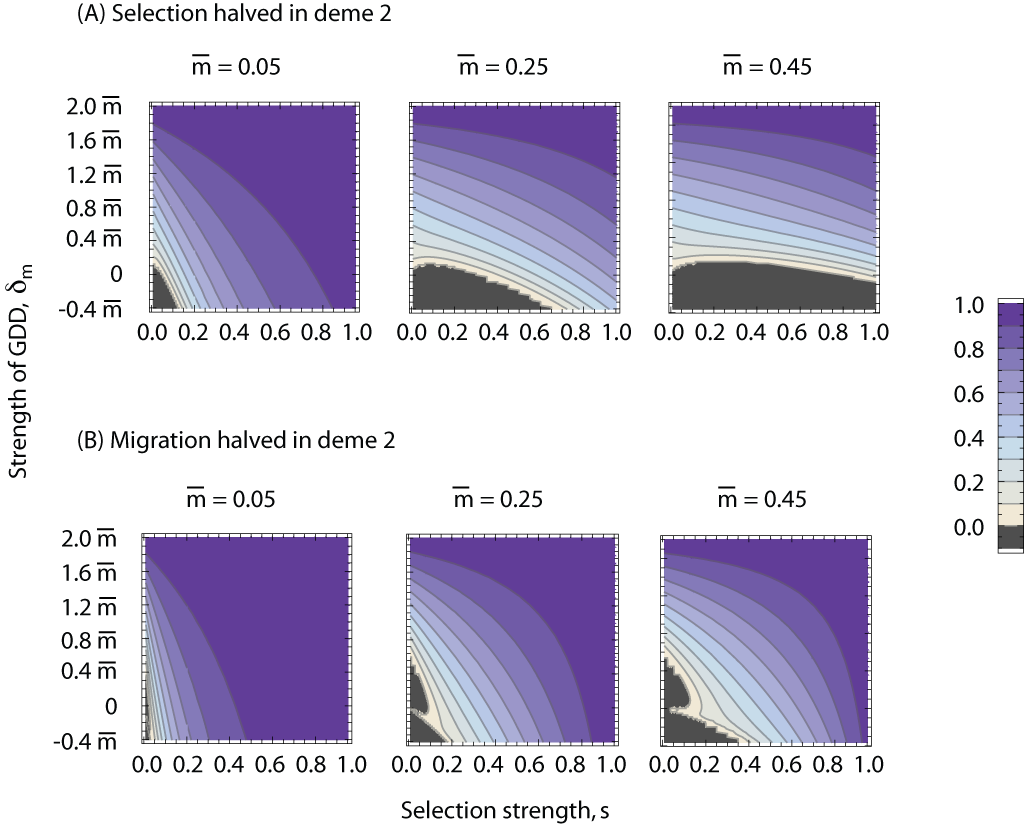


**Figure S4.** Degree of population divergence in allele frequency, $\Delta_{eq}$, with asymmetric selection (A) or migration (B) in the two habitats. Fig. S4 is identical to Fig. 2A, except that the strength of selection or migration is allowed to differ between the two patches: (A) Selection is half as strong in deme B (*s_A_* = *s* and *s_B_* = s/2). (B) Migration out of deme B is half as frequent ( and /2). Shading describes the amount of adaptive divergence in allele frequency at equilibrium, $\Delta_{eq}$, with grey representing regions of the parameter space where there is no local adaptation (either a polymorphism cannot be maintained and/or there is sufficient maladaptive dispersal that local adaptation does not occur ). As in the symmetric case in Fig. 2A, increasing adaptive dispersal (higher $\delta_{m}$) facilitates the maintenance of genetic polymorphism and promotes local adaptation.

**
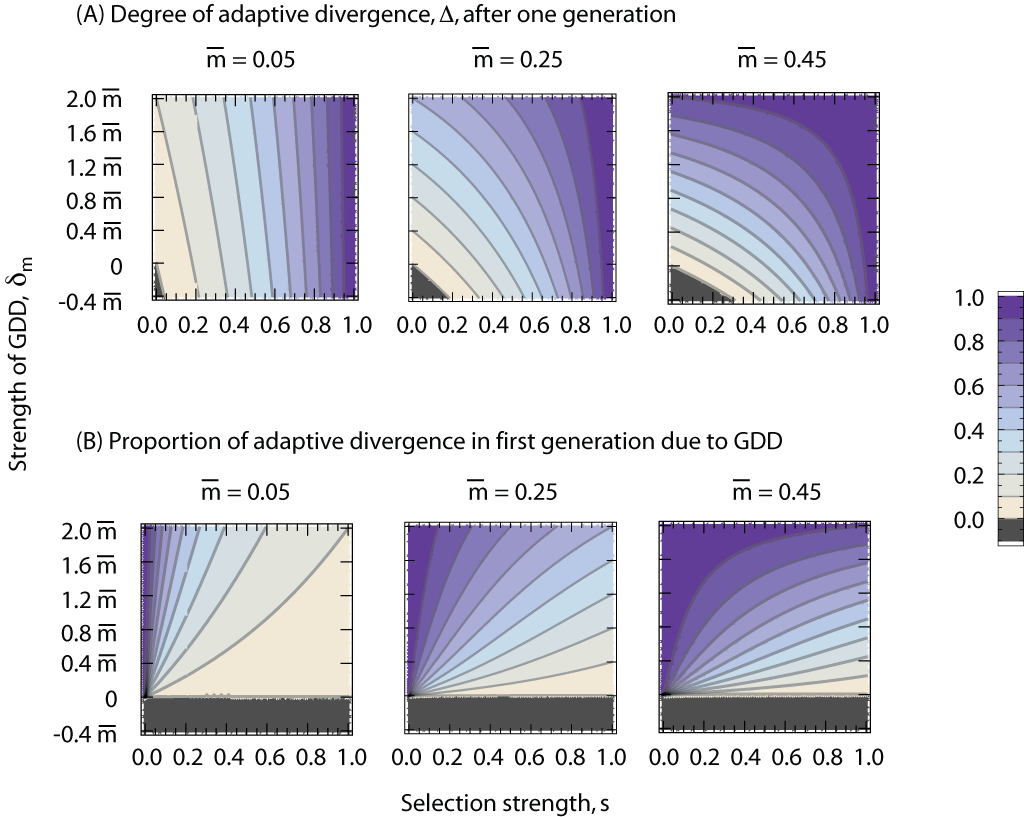
**

**Figure S5.** Amount of first-generation divergence in allele frequency due to GDD. (A) Top panels show the amount of divergence in allele frequencies between populations after one generation, $\Delta_{1}$, as the extent of adaptive habitat choice is increased ($\delta_{m}$, vertical axis) for a given strength of selection (horizontal axis) and migration (different panels). Populations are initially well mixed with allele frequencies equal to 1/2 everywhere ($\Delta_{0}=0$). (B) Bottom panels show the proportion of this one-generation response that is attributable to GDD and would be absent if all individuals migrated at equal rates (equation 13).


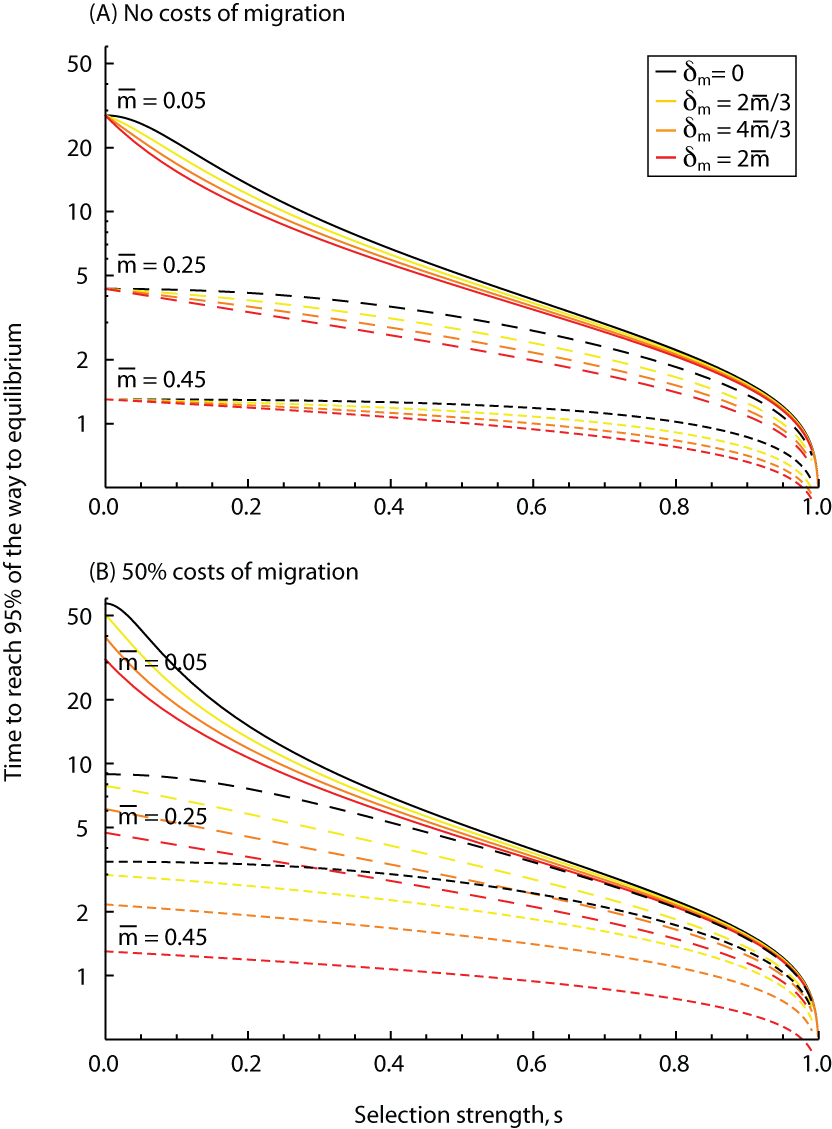


**Figure S6.** Time to approach equilibrium. Shown is the number of generations required for the system to move 95% of the way to equilibrium (α = 0.95). (A) No costs of migration. (B) 50% costs of migration (*c* = 0.5). Average migration rates are 0.05 (top solid curves), 0.25 (middle long-dashed curves), and 0.45 (bottom short-dashed curves). Color denotes the strength of GDD. Increasing the extent of adaptive habitat choice reduces the time taken to reach equilibrium (black: no GDD through to red: maximal GDD with $\delta_{m}=2\bar{m}$).

**Figure S7.** Adaptive dispersal increases allele frequency divergence between habitats. The figure is identical to Fig. 3 in the main text, except that the population begins with the *a* allele at a frequency of 0.05 in both habitats. Parameters: = 0.2, *s_A_* = *s_B_* = 0.4, $\delta_{m}=0.342$ (equivalent to θ = 5 under the specific model of habitat choice, equation 4).

**Figure S8.**  The effective migration rate between habitats changes dynamically with GDD. The two panels present the proportion of individuals that migrate from habitat *A* to *B* (left) and from *B* to *A* (right) starting with allele *a* at frequency 0.05 (as in Fig. S7). Initially, most individuals in habitat *A* carry the maladapted allele *b*, and so a higher fraction of individuals depart to habitat *B* than expected based on the average migration rate of the two alleles ( = 0.2). As the frequency of allele *a* rises in habitat A and individuals become more locally adapted, the emigration rate with adaptive dispersal declines (green: $\delta_{m}=0.342$), until it is much lower than expected under random dispersal (blue: $\delta_{m}=0$). Conversely, in habitat *B* the majority of individuals are well adapted at the outset of the simulation, and so emigration remains lower with GDD than without GDD throughout the simulation. Parameters: *s_A_* = *s_B_* = 0.4, $\delta_{m}=0.342$ is equivalent to θ = 5 under the specific model of habitat choice (equation 4).

**Figure S9.** Genotype-dependent dispersal causes emigrating individuals to be a biased sample of the individuals within a habitat. We illustrate this point by tracking the genotype frequencies within the two populations, and of migrants, over the course of the simulation presented in Fig. S7. Thick green lines represent the allele frequencies of individuals born in habitat A (solid line) and B (dotted line) that are available to disperse, as also represented by green lines in Fig. S7. Black lines show the allele frequencies of individuals that actually do disperse from habitat A to B (circles) or B to A (triangles). Differences between the allele frequency of dispersing individuals (black) and individuals born within a patch (green) indicate genotype-biased emigration caused by GDD. The frequency of allele *a* is always lower among emigrants from patch *A*, where *a* is favored, and higher among emigrants from patch *B*, where *a* is disfavored. Parameters: = 0.2, *s_A_* = *s_B_* = 0.4, $\delta_{m}=0.342$ (equivalent to θ = 5 under the specific model of habitat choice, equation 4).


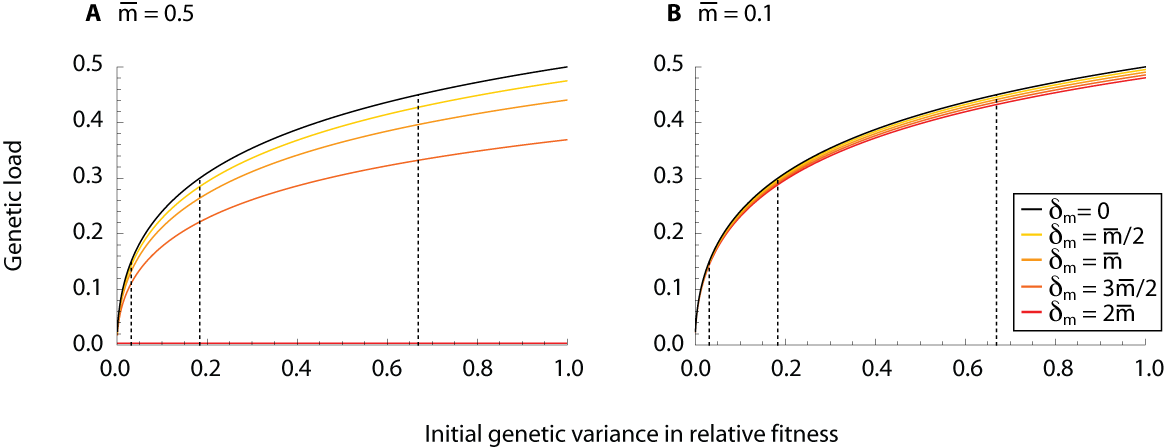


**Figure S10.** The relationship between variance in fitness at the start of a generation and the resulting genetic load, for two mean migration rates (A, B). This figure is identical to Fig. 4, except that the starting frequency of allele *a* is *p*_0_ = 0.05 in both patches. Again, GDD reduces the load in the generation, but the effect is less substantial when the initial allele frequency is lower, especially when the average migration rate is also low (right panel). Note that GDD fully eliminates the load with complete adaptive dispersal (red curve lies along the horizontal axis in panel A). Parameters: (A) = 0.5, (B) = 0.1, and *s* varying from 0 to 1 (the three dashed vertical lines correspond to *s* = 0.3, 0.6, and 0.9, from left to right).
